# Single-Cell and Bulk Transcriptome Data Integration Reveals Dysfunctional Cell Types and Aberrantly Expressed Genes in Hypertrophic Scar

**DOI:** 10.3389/fgene.2021.806740

**Published:** 2022-01-03

**Authors:** Shunuo Zhang, Yixin Zhang, Peiru Min

**Affiliations:** Department of Plastic and Reconstructive Surgery, Shanghai Ninth People’s Hospital Affiliated to Shanghai Jiao Tong University School of Medicine, Shanghai, China

**Keywords:** hypertrophic scar, extracellular matrix, single-cell RNA sequencing, cell-cell communication, myofibroblast, keratinocyte

## Abstract

Hypertrophic scar (HS) is a common skin disorder characterized by excessive extracellular matrix (ECM) deposition. However, it is still unclear how the cellular composition, cell-cell communications, and crucial transcriptionally regulatory network were changed in HS. In the present study, we found that FB-1, which was identified a major type of fibroblast and had the characteristics of myofibroblast, was significantly expanded in HS by integrative analysis of the single-cell and bulk RNA sequencing (RNA-seq) data. Moreover, the proportion of KC-2, which might be a differentiated type of keratinocyte (KC), was reduced in HS. To decipher the intercellular signaling, we conducted the cell-cell communication analysis between the cell types, and found the autocrine signaling of HB-1 through COL1A1/2-CD44 and CD99-CD99 and the intercellular contacts between FB-1/FB-5 and KC-2 through COL1A1/COL1A2/COL6A1/COL6A2-SDC4. Almost all the ligands and receptors involved in the autocrine signaling of HB-1 were upregulated in HS by both scRNA-seq and bulk RNA-seq data. In contrast, the receptor of KC-2, SDC4, which could bind to multiple ligands, was downregulated in HS, suggesting that the reduced proportion of KC-2 and apoptotic phenotype of KC-2 might be associated with the downregulation of SDC4. Furthermore, we also investigated the transcriptionally regulatory network involved in HS formation. The integrative analysis of the scRNA-seq and bulk RNA-seq data identified CREB3L1 and TWIST2 as the critical TFs involved in the myofibroblast of HS. In summary, the integrative analysis of the single-cell RNA sequencing (scRNA-seq) and bulk RNA-seq data greatly improved our understanding of the biological characteristics during the HS formation.

## Introduction

Hypertrophic scar (HS) caused by pathologically excessive collagen deposition from skin fibroblasts in the dermis and subcutis are major types of pathological scars that can be regarded as complications to abnormal wound healing (Haverstock, 2001). Unlike keloids, HS does not extend beyond the borders of the original wound, as myofibroblasts, the main effector cell contributing to dermal fibrosis, are present in hypertrophic scarring, and α-smooth muscle actin (α-SMA) is expressed in a nodular formation in the fibroblasts from HS (Lee et al., 2004; Feng et al., 2020). Meanwhile, abnormal keratinocyte differentiation and proliferation, and significantly increased acanthosis can be observed along with hypertrophic scarring (Niessen et al., 2001).

Through animal models, it is found that inflammatory cells, bone marrow-derived fibrocytes, and several peptide-related compounds may serve as essential players in hypertrophic scar development (Satoyoshi, 1966; Song et al., 2020; Wang et al., 2020). It has been well-recognized that the expressions of keratinocyte-derived interleukin-1 (IL-1), tumor necrosis factor-a (TNF-a), platelet-derived growth factor (PDGF), transforming growth factor-b (TGF-b) and basic fibroblast growth factor (bFGF) are associated with extracellular matrix (ECM) remodeling, and medicines or potential reagents to prevent and treat hypertrophic scars could result in inflammatory inhibition via targeting these molecules (Limandjaja et al., 2018; Wang et al., 2020). A previous research has described that fibrocytes, functioning as antigen-presenting cells, may contribute to the upregulation of the inflammatory response, and another study has demonstrated that the TGFβ1/Smad pathway could regulate ECM synthesis via stimulating fibroblasts and inducing fibroblast differentiation into myofibroblasts, thus promoting the formation of hypertrophic scars (Yang et al., 2005; Liu et al., 2012; Shirakami et al., 2020). Also, activation of PI3K/AKT pathway could lead to hypertrophic scar formation and ECM deposition via upregulated expression of Collagen I, Collagen III, α-SMA, and Cleaved caspase-3 in hypertrophic scar fibroblasts (Xiao, 2020; Zhi et al., 2021). In addition, some transcription factors (TFs) such as CRBE3L1 and TWIST2 have been identified as critical regulators in skin diseases (Crespo et al., 2021; Deng et al., 2021). Importantly, the two TFs were also involved in kidney fibrosis (Grunz-Borgmann et al., 2017; Yamamoto et al., 2021). However, few hypertrophic scar animal models could perfectly reflect human skin injuries, and though much effort has been dedicated to the study of abnormal wound healing, the pathogenesis of hypertrophic scarring has not been fully unveiled, and breakthrough development in the therapeutic management for hypertrophic scars is urgently needed, as hypertrophic scarring would lead to considerable morbidity (Wang et al., 2011; Domergue et al., 2015).

Utilization of single-cell RNA sequencing (scRNA-seq) has helped examine cell variability across tumors and interactions between detected cell types (Kumar et al., 2018), and with the aid of scRNA-seq technologies, exploring fibroblast heterogeneity at a single-cell resolution and cell-cell communication in the context of hypertrophic scarring has now become a reality. Recent study identifies serine proteases as regulators of myofibroblast differentiation using single cell sequencing technology (Vorstandlechner et al., 2020). In the present study, we hope to identify potential cell-cell communications and abnormal transcriptionally regulatory network in hypertrophic scarring.

## Materials and Methods

### Data Collection

The scRNA-seq data of normal and HS samples were collected from Gene Expression Omnibus (GEO) with accession number GSE156326 (Vorstandlechner et al., 2020). The bulk RNA sequencing data was generated by this study. The five normal and five HS skin tissue samples were collected from Nineth People’s Hospital of Shanghai Jiao Tong University, School of Medicine, which was approved by the Human Research Ethics Committee of this hospital. The written informed consents were collected from each patient. All samples were stored in −80°C for the following experiments.

### RNA Extraction, Library Construction and RNA Sequencing Analysis

RNA extraction and sequencing was performed at the Beijing Genomics Institute (BGI), using an Illumina HiSeq 4,000 sequencer (Illumina) following the standard manufacturer’s protocols as described by the previous study (Hillen et al., 2019). The raw data was first preprocessed by excluding the reads with low quality. The clean reads were the mapped to human reference genome (GRCh37/hg19) using Hisat v2.2.1 (Kim et al., 2015). The gene expression quantification was performed by using Stringtie v2.1.4 (Pertea et al., 2015) and R ballgown package (Frazee et al., 2015). The default parameters of Hisat and Stringtie were used in this study. The count table was generated by the python script prepDE.py (https://ccb.jhu.edu/software/stringtie/dl/prepDE.py) from Stringtie. The R/Bioconductor DESeq2 (Love et al., 2014) package was applied to differential gene expression analysis.

### Cell Clustering Analysis

The unique molecular identifiers (UMIs) count-based scRNA-seq data of six human skin samples from GEO accession GSE156326 were used for the cell clustering analysis, which was implemented in R Seurat package. Cells with less than 200 UMIs were eliminated and features detected in less than three cells were filtered. The multiple datasets were integrated by SCTransform in Seurat package. The top 3,000 highly variable features were selected by FindVariableFeatures. The clusters were found at a resolution of 0.8 by FindClusters, and T-distributed Stochastic Neighbor Embedding (t-SNE) was applied to reduce the dimensionality. The cell-type marker genes were detected by FindAllMarkers function at adjusted *p*-value < 0.05, minimal percentage difference >0.25, and log2 fold change >0.5. All the marker genes of the cell clusters were collected from the earlier study (Vorstandlechner et al., 2020). This analysis was implemented by R Seurat package (Stuart et al., 2019).

### Estimation of Cell Proportion

The cell proportions were estimated using MuSiC(Wang et al., 2019), which used a deconvolution method based on marker genes of cell types and gene expression matrices of both scRNA-seq and bulk RNA-seq to estimate the cell proportions of bulk RNA-seq data. The count-based expression data of both scRNA-seq and bulk RNA-seq was applied to this analysis.

### Gene Set Enrichment Analysis (GSEA)

The GSEA was conducted against KEGG, Reactome (Jassal et al., 2020), WikiPathway, and Gene Ontology (GO-bp). The GSEA was implemented in R clusterProfiler (Yu et al., 2012).

### Cell-Cell Communication Analysis

The cell-cell communication was predicted by R CellChat (Jin et al., 2021) package. The normalized count and cell types by Seurat were used for this analysis. This analysis was separately conducted on the normal and HS cells.

### Transcriptionally Regulatory Network Analysis

The transcriptionally regulatory network analysis was performed by R SCENIC package (Aibar et al., 2017). The AUCell values for the transcription factors (TFs) were used for differential analysis (Aibar et al., 2017).

### Statistical Analyses

All the statistical analyses were performed in R (version 4.1.0). The two-sample and pairwise comparisons were conducted by Wilcoxon rank sum test. The symbols of *, **, and *** represent the statistical significance at 0.05, 0.01, and 0.001, respectively.

## Results

### Single-Cell and Bulk RNA-Seq Reveal the Cellular Diversity and Heterogeneity of Skin Tissues.

**FIGURE 1:**
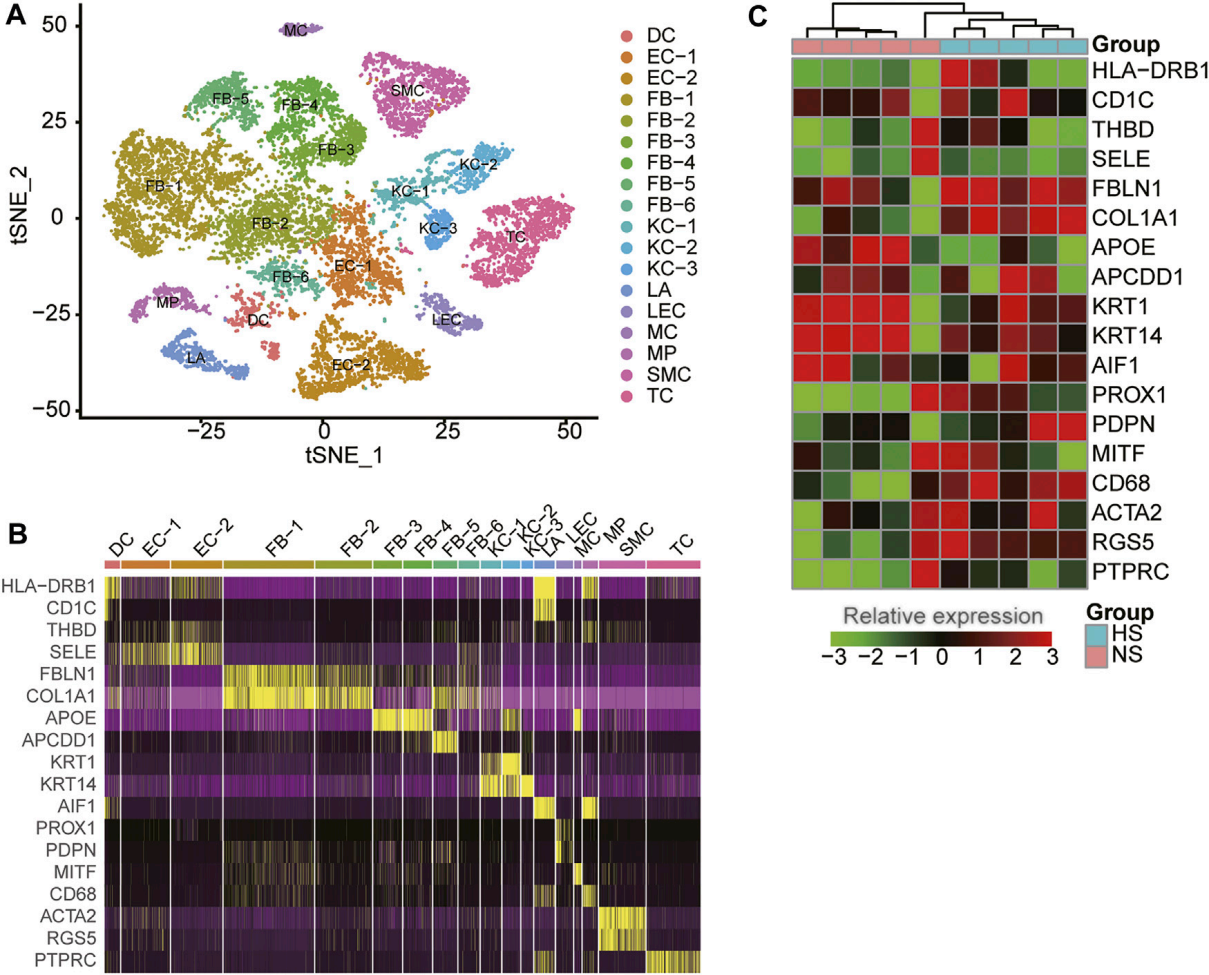
The cell populations and marker genes in the skin samples of hypertrophic scar (HS) and normal skins. **(A)** The cell clusters visualized by the dimensional reduction of t-distributed stochastic neighbor embedding (t-SNE). Each point represents one cell, and the filled colors represent the cell types. **(B)** The expression specificity of cell type-specific marker genes within the skin samples using scRNA-seq data. **(C)** The differential expression of the cell type-specific marker genes between HS and normal skins using bulk RNA-seq data.

To reveal the cellular diversity and heterogeneity of skin tissues, we collected a single-cell RNA sequencing (RNA-seq) dataset of three normal (NS) and three hypertrophic scar (HS) samples from Gene Expression Omnibus (GEO) database (See Materials and methods). After excluding the cells with low quality, we identified 18 cell clusters from 15,276 cells by the dimensional reduction and clustering analysis (Figure 1A), and characterized the cell clusters using the marker genes from previous studies (Solé-Boldo et al., 2020; Vorstandlechner et al., 2020; Liu et al., 2021). The cell clusters included six clusters of fibroblasts (FB-1/2/3/4/5/6, represented by marker genes: FBLN1, COL1A1, APOE, and APCDD1), three clusters of keratinocytes (KC-1/2/3: KRT1 and KRT14), two clusters of endothelial cells (EC-1/2: THBD and SELE), T cells (TC: PTPRC), smooth muscle cells (SMC: ACTA2 and RGS5), Langerhans cells (LA: AIF1), lymphatic endothelial cells (LEC: PROX1 and PDPN), dendritic cells (DC: HLA−DRB1 and CD1C), macrophage (MP: CD68), and melanocyte (MC: MITF) (Figure 1B), suggesting that the cell types were well characterized by the marker genes.

To reveal the expression patterns of the cell-type marker genes in skin tissues, we also collected five NS and five HS tissue samples for bulk RNA sequencing. As shown in Figure 1C, the representative marker genes were found to be differentially expressed between the NS and HS tissues (adjusted *p*-value < 0.05 and fold change >1.5). Moreover, about 26.83% of the cell-type marker genes (376/1,401) were observed to be differentially expressed between NS and HS samples. The deregulation of the cell-type marker genes in HS samples indicated that the cell proportions and gene expression patterns were changed in HS.

### Differential Proportion Analysis Reveals Significant Expansion of Fibroblast and Contraction of KC Subpopulations in HS

**FIGURE 2:**
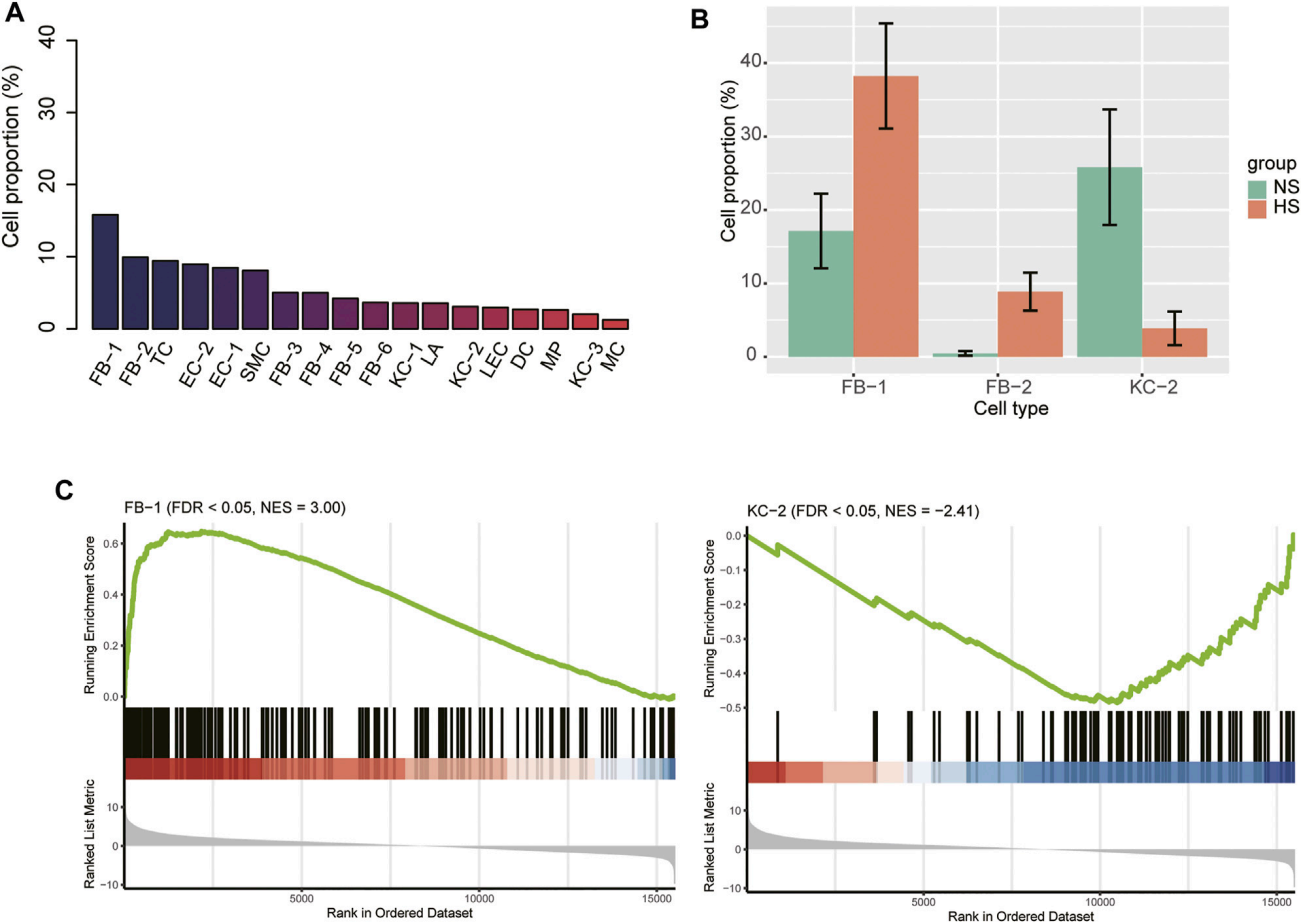
The cell proportions within HS and normal skin samples. **(A)** The cell proportions within the integrated HS and normal skin samples. **(B)** The cell types with differential proportions between HS and normal skin. **(C)** The differential expression levels of the FB-1 and KC-2 specific marker genes between the HS and normal skin samples.

We next attempted to identify HS-associated cell lineages or clusters that were significantly expanded or contracted in HS. The FB-1 accounted for the largest number of cells, followed by FB-2, TC, and EC-2 based on the single-cell RNA-seq data (Figure 2A). Furthermore, we also conducted gene set enrichment analysis on the marker genes of those cell types to characterize their functionalities (Supplementary Table S1). Remarkably, the three major cell types, including FB-2, EC-2, and T cell, were characterized by collagen degradation, VEGFA-VEGFR2 Signaling Pathway, and TYROBP Causal Network, respectively. The cell proportions of the three NS and three HS samples could be estimated by the single-cell RNA-seq data. To improve the reliability of the differential proportion analysis, we also estimated the cell proportions for the ten bulk RNA-seq samples using MuSiC (Wang et al., 2019), a deconvolution method to estimate cell type proportions from bulk RNA sequencing data. The differential proportion analysis revealed that FB-1 and FB-2 were expanded in HS, while the proportion of KC-2 was reduced in HS (Figure 2B). Accordingly, the FB-1 and KC-2 marker genes were enriched in the up- and down-regulated genes in HS by bulk RNA-seq data (Figure 2C), respectively. However, FB-2 marker genes were enriched in neither the upregulated nor the downregulated genes in HS. These results suggested that the changed proportions of these cell types might be closely associated with HS.

### Fibroblast Heterogeneity in Skin Tissues

**FIGURE 3:**
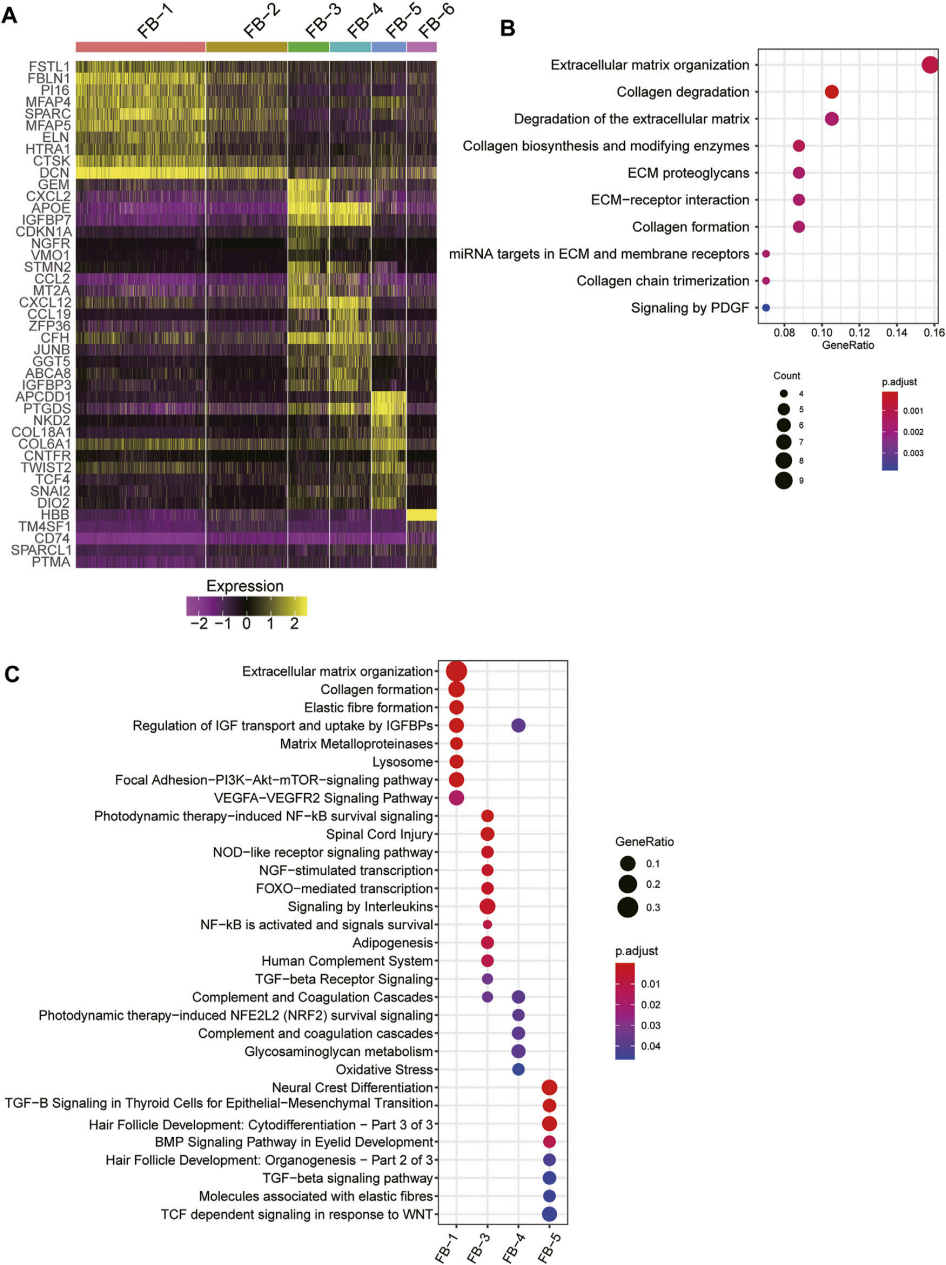
The fibroblast subpopulations in the skin samples. **(A)** The expression specificity of the FB subpopulation specific genes across the FB cell populations. **(B)** The upregulated pathways in FB-1 cells of HS. **(C)** The subpopulation specific biological functions across the FB subpopulations.

As fibroblast clusters were identified as the major cell types in skin tissues by scRNA-seq data, we then investigated their functional differences by comparing their expression profiles. The differential gene expression analysis revealed that FB-1, FB-3, FB-4, and FB-5 had their top-ten specific marker genes, while no marker genes specifically upregulated in FB-2 or FB-6 were identified (Figure 3A). Consistent with the excessive ECM deposition observed in bulk RNA-seq data of HS (Figure 3B), ECM-related pathways, such as extracellular matrix organization, elastic fiber formation, collagen formation, and matrix metalloproteinases, and PI3K-Akt signaling were significantly upregulated in FB-1 (Figure 3C). FB-3 was characterized by the inflammatory pathways, such as photodynamic therapy-induced NF-kB survival signaling, NOD-like receptor signaling pathway, and signaling by interleukins (Figure 3C). The oxidative stress and Wnt signaling were identified as the signature pathway of FB-4 and FB-5 (Figure 3C), respectively. These results indicated that fibroblasts were functionally heterogenous in skin tissues.

**FIGURE 4:**
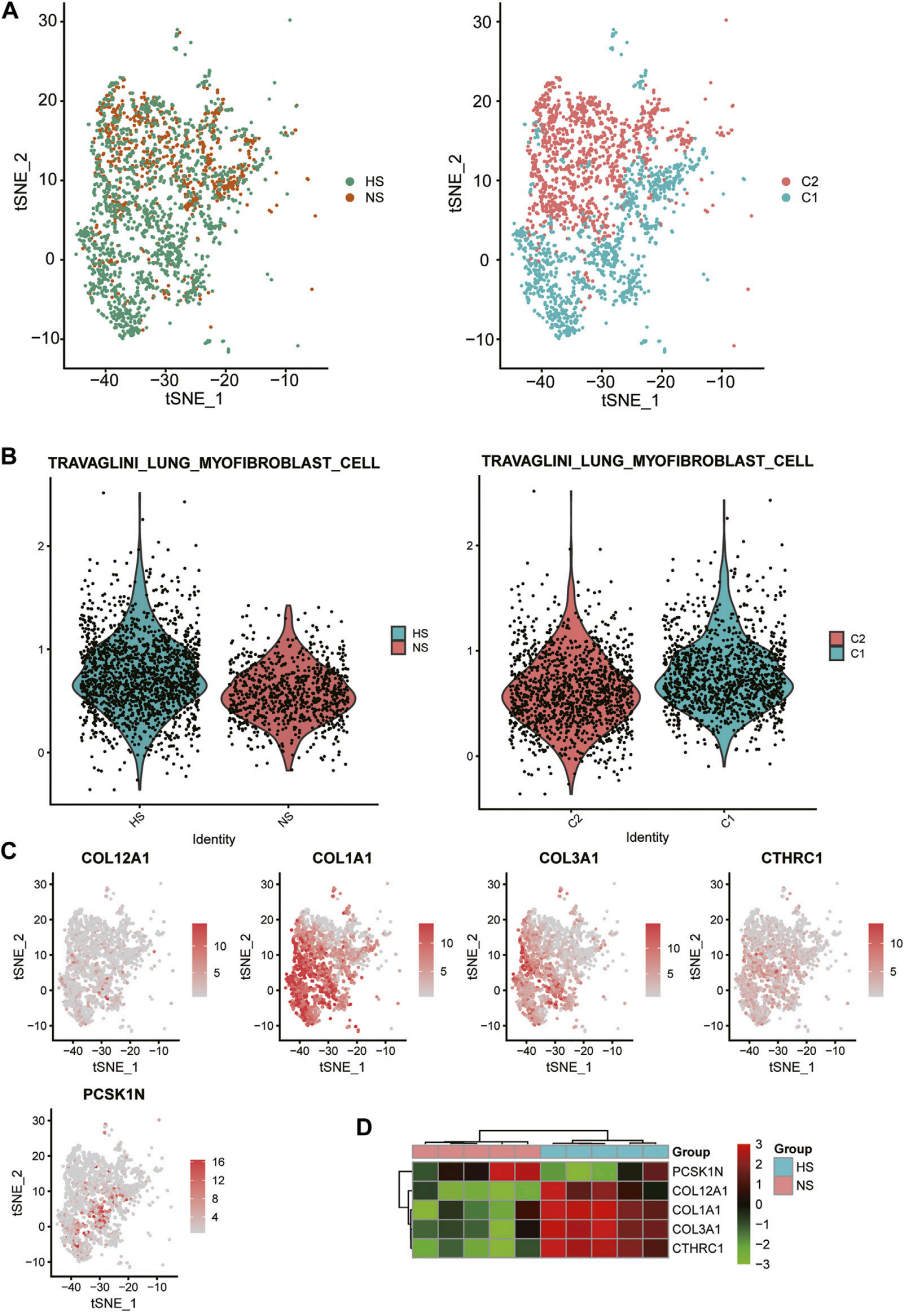
Biological function of HS-related FB-1 cluster. **(A)** The clustering of the HS and normal skin (NS)-related FB-1 cells grouped by disease (HS vs NS) and graph-based classification (C1 vs C2), respectively. **(B)** The differential module score of myofibroblast cells between HS and NS, and C1 and C2. **(C)** The expression levels of myofibroblast cell marker genes including COL12A1, COL1A1, COL3A1, CTHRC1, and PCSK1N in the HB-1 cells. **(D)** The differential expression levels of the myofibroblast cell marker genes between HS and NS bulk samples.

As FB-1 was one of the major cell types expanded in HS, we then investigated the biological function of this FB type. Interestingly, the FB-1 cells from HS samples could be clearly distinguished from those from NS samples (Figure 4A). The graph-based clustering analysis revealed that the C1 subpopulation was significantly enriched by the cells from HS (Figure 4A, hypergeometric test, *p*-value < 0.05). Moreover, we also found that the myofibroblast gene signatures were higher in C1 and HS, as compared with C2 and NS cells (Figure 4B), respectively, suggesting that the FB-1 from HS might have a myofibroblast phenotype. Moreover, the myofibroblast signature genes, including COL12A1, COL1A1, COL3A1, CTHRC1, and PCSK1N, were expressed higher in C1 than C2 (Figure 4C). The bulk RNA-seq data further confirmed the upregulation of the five genes except PCSK1N (Figure 4D). Notably, the five genes were involved in collagen formation, which is a critical regulator of myofibroblast differentiation (Yuen et al., 2010). These results indicated that HS formation was associated with both the increased proportion and the biological characteristics of myofibroblast.

### Dysregulation of Keratinocytes Signature Genes in HS

**FIGURE 5:**
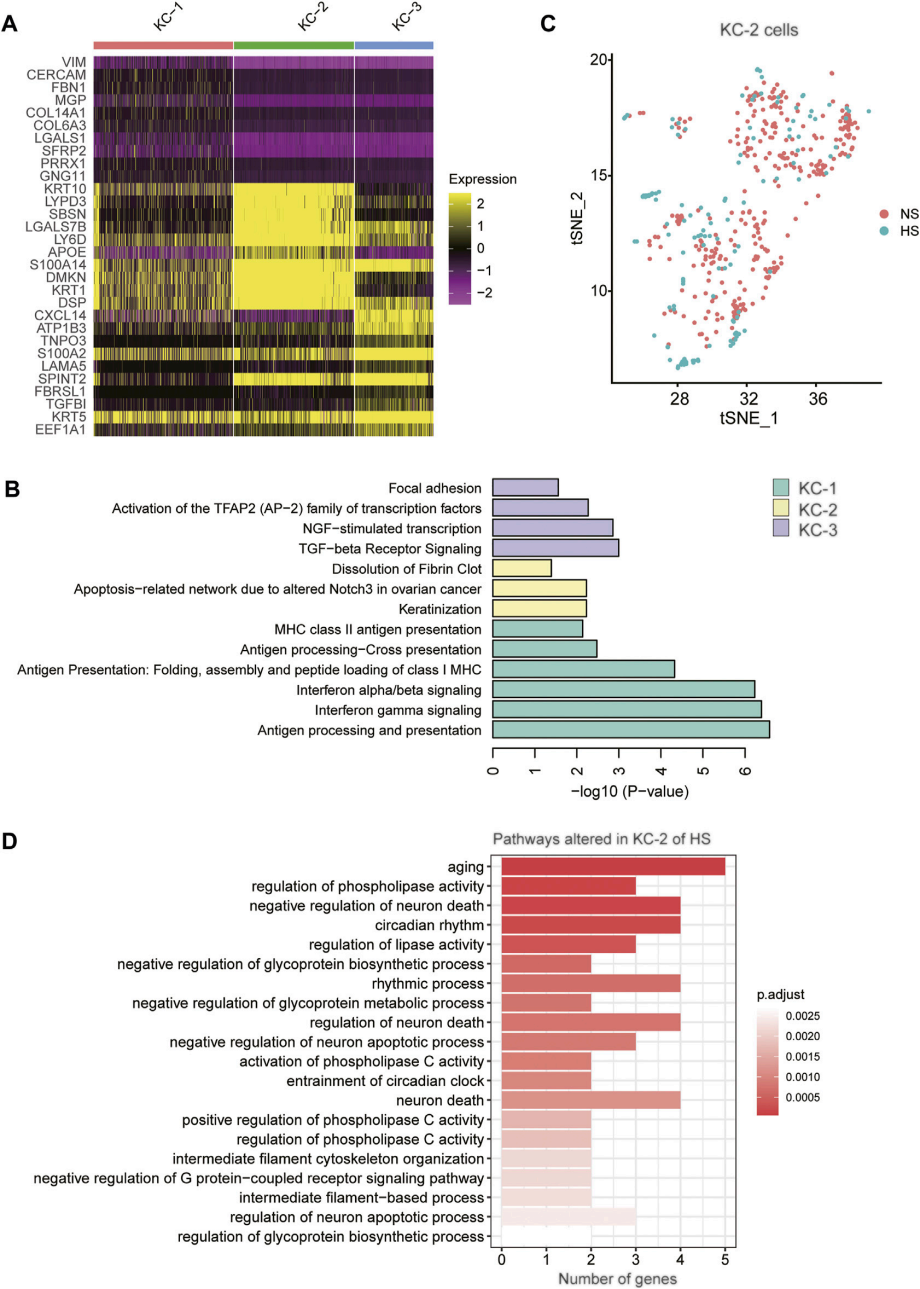
The keratinocyte subpopulations in the skin samples. **(A)** The expression specificity of the KC subpopulation specific genes across the KC cell populations. **(B)** The subpopulation specific biological functions across the KC subpopulations. **(C)** The clustering of the KC-2 cells grouped by disease (HS vs NS). **(D)** The representative biological functions in KC-2 cells of HS.

As the proportion of type 2 keratinocyte was reduced in HS, we then asked whether KC-2 in HS showed distinct expression patterns relative to those in NS. Compared to KC-1 and KC-3, the KC-2 exerted distinct expression profiles (Figure 5A). The gene set enrichment analysis revealed that the three KC cell types (KC-1/2/3) might be associated with antigen presentation, keratinization, and transforming growth factor-beta (TGFbeta) signaling (Figure 5B). The tSNE analysis revealed that the KC-2 in HS and NS could not be clearly distinguished (Figure 5C), suggesting that the KC-2 in HS and NS showed similar expression patterns. The differential gene expression analysis between KC-2 cells of HS and NS revealed that cell senescence or apoptosis-related pathways such as aging, neuron death, and regulation of neuron apoptotic process were upregulated in KC-2 cells of HS (Figure 5D), suggesting that the keratinocyte apoptosis might be associated with the reduced proportion of KC-2 in HS.

### Cell-Cell Communications

**FIGURE 6:**
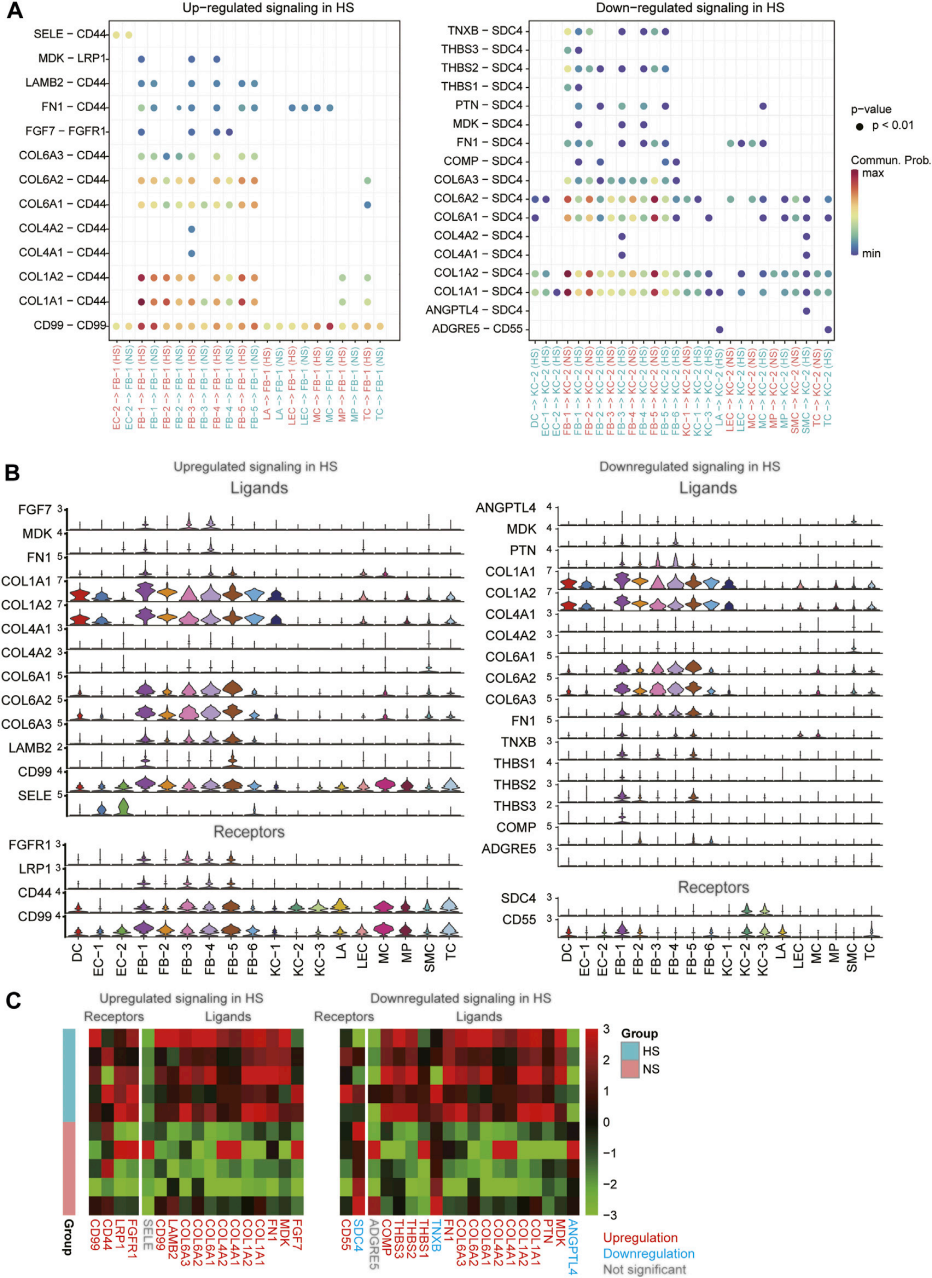
The differential cell-cell communications between HS and NS. **(A)** The upregulated and downregulated signaling in HS. The x- and *y*-axis represent the cell-cell communications and ligand-receptor pairs, respectively. **(B)** The expression levels of the ligands and receptors involved in the upregulated or downregulated signaling across the cell types. **(C)** The differential expression levels of the ligands and receptors between the HS and NS bulk RNA-seq samples.

To decipher the intercellular signaling, we conducted the cell-cell communication analysis between the cell types using CellChat (Jin et al., 2021). As the FB1-1 and KC-2 exhibited dominant functional roles in HS formation, we then investigated the signaling sources of the two cell types. Specifically, we identified 13 ligand-receptor (LR) pairs upregulated in FB-1 of HS and 17 LR pairs downregulated in KC-2 of HS (Figure 6A, adjusted *p*-value < 0.05). Particularly, we found that the autocrine signaling of HB-1 through COL1A1/2-CD44 and CD99-CD99 achieved the high communication probabilities (Figure 6A). Similarly, the cell-cell contacts between FB-1/FB-5 and KC-2 through COL1A1/COL1A2/COL6A1/COL6A2-SDC4 had the high communication probabilities in the downregulated cell-cell communication network (Figure 6A). The expression analysis revealed that the ligands and receptors such as COL1A1, COL1A2, CD44, and CD99 involved in the signaling transduction of FB-1 had higher expression levels in FB-1, as compared with the other cell types (Figure 6B). In contrast, the receptors involved in the signaling transduction of KC-2 were specifically expressed in KC-2 and KC-3, and the ligands were more specifically expressed in FB-1 and FB-5 (Figure 6B). Moreover, we also investigated the differential expression levels of those ligands and receptors between the HS and NS using the bulk RNA-seq data. Notably, all the ligands and receptors involved in signaling transduction of FB-1 except SELE were found to be upregulated in HS (Figure 6C). Interestingly, the receptor SDC4, which could bind to multiple ligands, were downregulated in HS, suggesting that the reduced proportion of KC-2 and apoptotic phenotype of KC-2 might be associated with the downregulation of SDC4.

### Transcriptionally Regulatory Network Involved in HS Formation

**FIGURE 7:**
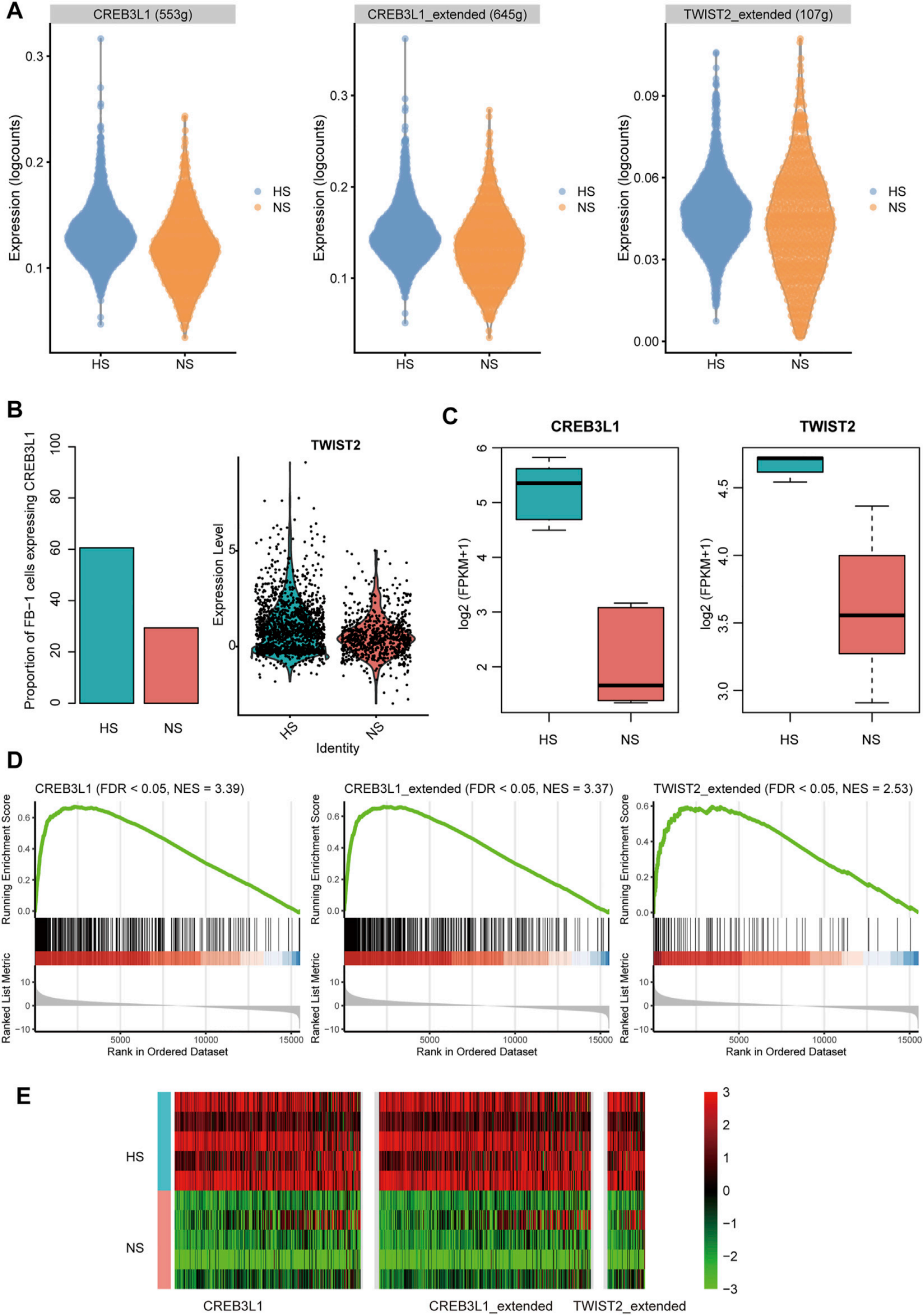
The transcription factors (TFs) and regulatory network involved in HS. **(A)** The differential transcriptional activities of the key TFs in FB-1 of HS samples. **(B)** The differential proportion of FB-1 cells expressing CREB3L1 and differential expression of TWIST2 between the FB1- cells of HS and NS. **(C)** The differential expression of TFs between the bulk RNA-seq samples of HS and NS. **(D,E)** Upregulation of TF target genes in the bulk RNA-seq samples of HS by gene set enrichment analysis (GSEA).

To infer the transcriptionally regulatory network involved in HS formation, we estimated the transcriptional activities of the transcription factors (TFs) using SCENIC(Aibar et al., 2017). The differential transcriptional activity analysis revealed that the FB-1 cells in HS had higher transcriptional activities of CREB3L1 and TWIST2 than those in NS (Figure 7A). Unfortunately, we did not observe any TFs which showed different activities between HS and NS of KC-2. HS had a higher proportion of the FB-1 cells expressing CREB3L1 than NS as CREB3L1 was lowly expressed in FB-1 (Figure 7B). The FB-1 in HS had a higher expression level of TWIST2 than that in NS (Figure 7B, adjusted *p*-value < 0.05). The previous study identified JUN as a critical regulator by promoting hypertrophic skin scarring (Griffin et al., 2021). Consistently, we also found that the transcriptional activity of JUN was higher in HS than NS (Supplementary Figure S1).

Furthermore, we also investigated the expression levels of the TFs and target genes in HS and NS samples using bulk RNA-seq data. Consistently, the two TFs, CREB3L1 and TWIST2, were upregulated in HS as compared with NS using the bulk RNA-seq data (Figure 7C, *p*-value < 0.01). In according with the finding using scRNA-seq data, the target genes of CREB3L1 or TWIST2 also gathered in the upregulated genes in HS (Figures 7D,E, FDR <0.05). Similarly, the target genes of the two TFs were also significantly overlapped with the upregulated genes in the HS samples from a previous study (GEO accession: GSE188952, Supplementary Figure S2), further indicating that the transcriptional activities of the two TFs were upregulated in HS. These results suggested that the two TFs, CREB3L1 and TWIST2, might play key roles in HS formation.

## Discussion

Hypertrophic scar (HS) is a fibroproliferative skin disorder characterized by excessive extracellular matrix (ECM) deposition. However, it is still unclear how the cellular composition, cell-cell communications, and crucial transcriptionally regulatory network were changed in HS. In the present study, we found that fibroblasts (FB) and keratinocytes (KC) had multiple cell clusters. The FB clusters were characterized by excessive ECM deposition, inflammatory, and proliferative signatures. Particularly, excessive ECM deposition, identified as the signature of FB-1, is associated with myofibroblast (Hinz and Lagares, 2020) and fibrosis (Agarwal et al., 2013). Moreover, the FB-1 was expanded in HS and FB-1 accounted for the largest proportion in the HS samples, suggesting that an increase of myofibroblast proportion in HS might be the major cause of the HS. The myofibroblast was considered as a therapeutic target of HS (Feng et al., 2020; Yang et al., 2020). The three KC cell types (KC-1/2/3) might be associated with antigen presentation, keratinization, and transforming growth factor-beta (TGF-beta) signaling. Remarkably, the proportion of KC-2, which might be a differentiated type of KC, was reduced in HS. The cell senescence or apoptosis-related pathways such as aging, neuron death, and regulation of neuron apoptotic process were upregulated in KC-2 cells of HS, suggesting that the keratinocyte apoptosis might be associated with the reduced proportion of KC-2 in HS. It has been well recognized that keratinocyte proliferation and migration are critical for re-epithelialization during cutaneous wound healing (DiPersio et al., 2016; Piipponen et al., 2020).

To decipher the intercellular signaling, we conducted the cell-cell communication analysis between the cell types, and found the autocrine signaling of HB-1 through COL1A1/2-CD44 and CD99-CD99 and the intercellular contacts between FB-1/FB-5 and KC-2 through COL1A1/COL1A2/COL6A1/COL6A2-SDC4. It has been reported that COL1A1/2 expression promotes the myofibroblast differentiation (Gambini et al., 2018), fibrosis (Kong et al., 2019), and HS formation (Bi et al., 2017). The receptor, CD44, was associated with an abnormal accumulation of extracellular matrix (Messadi and Bertolami, 1993). Moreover, CD44 was also identified as a mediator of myofibroblast differentiation and fibrosis (Woods et al., 2021). CD99, the other key receptor expressed in myofibroblast, was implicated in skin disorders (Kazakov et al., 2005; Belonogov and Ruksha, 2013). However, the intracellular signaling regulated by CD99 was still unclear. In contrast, the receptors involved in the signaling transduction of KC-2 were specifically expressed in KC-2 and KC-3, and the ligands were more specifically expressed in FB-1 and FB-5. Interestingly, the receptor SDC4, which could bind to multiple ligands, was downregulated in HS, suggesting that the reduced proportion of KC-2 and apoptotic phenotype of KC-2 might be associated with the downregulation of SDC4. Particularly, SDC4 could induce the keratinocyte activation (Bizzarro et al., 2019; Pessolano et al., 2019), which played a vital role in wound healing (Zhang et al., 2021). The low expression of SDC4 indicated that the signaling from fibroblasts could not be transduced to keratinocyte.

Furthermore, we also investigated the transcriptionally regulatory network involved in HS formation. The integrative analysis of the scRNA-seq and bulk RNA-seq data identified CREB3L1 and TWIST2 as the critical TFs involved in the myofibroblast of HS. Consistently, CRBE3L1 has been identified as one of the critical TFs involved in fibrotic skin diseases by a scRNA-seq study (Deng et al., 2021). The other TF, TWIST2, was relevant to Setleis syndrome (SS), a focal facial dermal dysplasia presenting with bilateral temporal skin lesions (Crespo et al., 2021). Moreover, TWIST1, an important paralog of TWIST2, was involved in the fibrogenesis of keloid fibroblasts, and might serve as a therapeutic target of keloid (Liu et al., 2021), suggesting that TWIST2 might also be considered as a promising therapeutic target in HS.

Even though cell-cell communications and key TFs involved in HS have been predicted based on the integrative analysis, there is still a lack of enough evidence to link the relationship between the transcriptionally regulatory network of CRBE3L1 or TWIST2 and the intercellular signaling like COL1A1/2-CD44 and CD99-CD99, which will be clarified by our future study.

In summary, the present study identified that an increase of myofibroblast proportion in HS, abnormal ligand-receptor interaction, and two key TFs might be promising therapeutic targets for HS. The integrative analysis of the scRNA-seq and bulk RNA-seq data not only identified key cell types altered in HS, but also shed light on the potential cell-cell communications and intracellular TF regulatory network involved in HS, which greatly improved our understanding of the biological characteristics during the HS formation.

## Data Availability

The datasets presented in this study can be found in online repositories. The names of the repository/repositories and accession number(s) can be found below: https://www.biosino.org/node/project/detail/OEP002674, OEP002674
