# Supplementary figures and images for: Single-Cell and Bulk Transcriptome Data Integration Reveals Dysfunctional Cell Types and Aberrantly Expressed Genes in Hypertrophic Scar
Source: Front Genet. 2022 Jan 3;12:806740. doi: 10.3389/fgene.2021.806740 (PMC8762316; doi:10.3389/fgene.2021.806740)

# JUN1

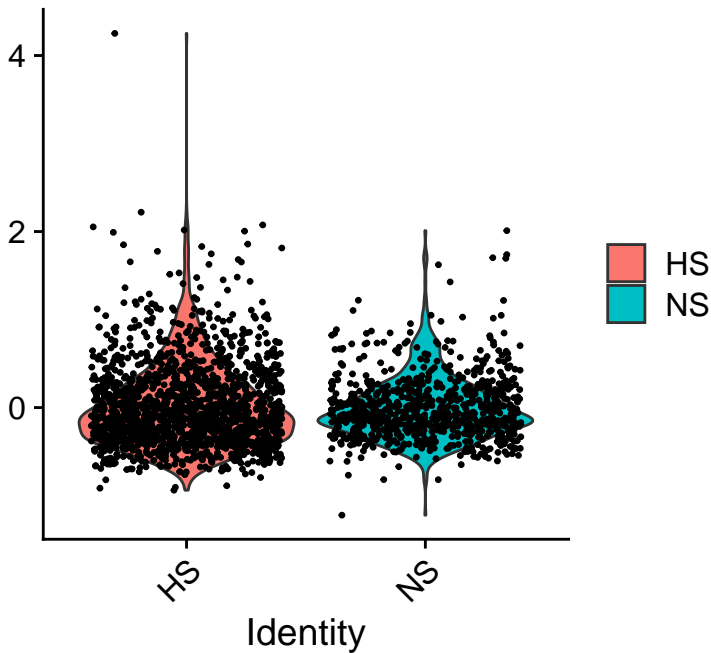

Supplement: Supplementary file 2 [file Presentation1.PDF]

CREB3L1 (FDR < 0.05, NES = 2.03)

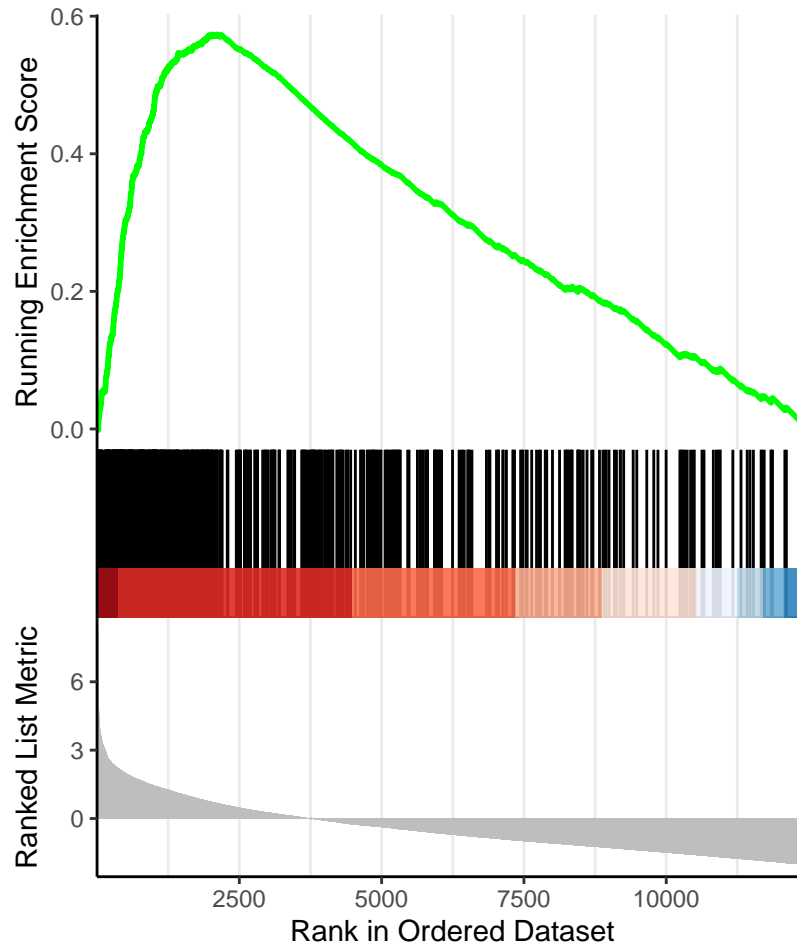

CREB3L1\_extended (FDR < 0.05, NES = 2.03)

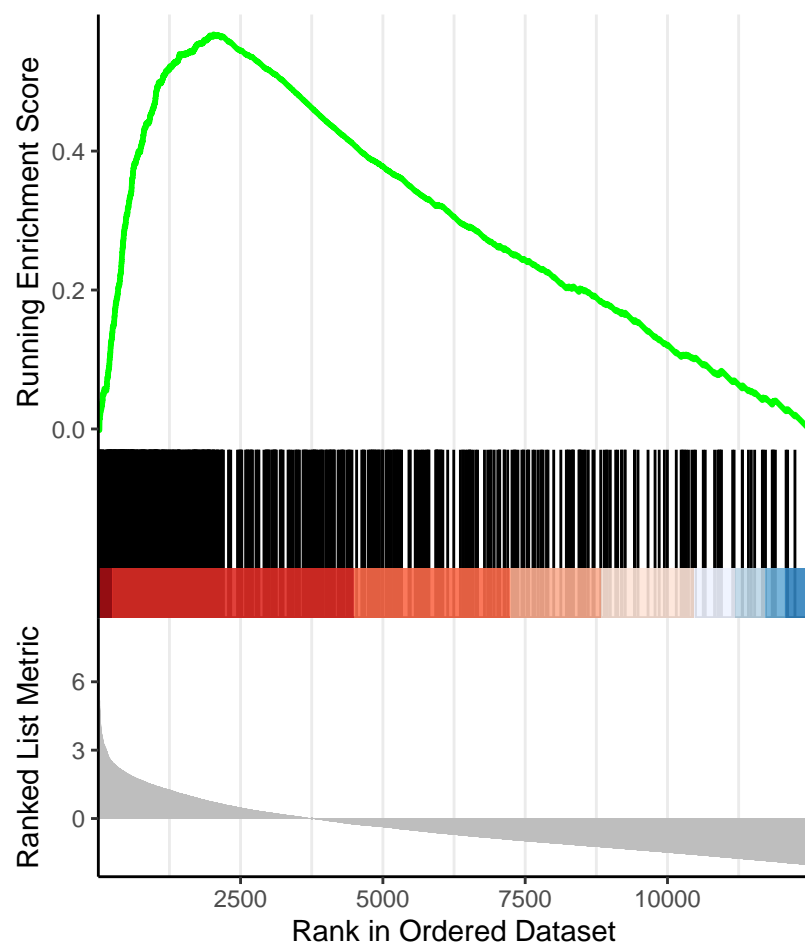

TWIST2 (FDR < 0.05, NES = 1.44)

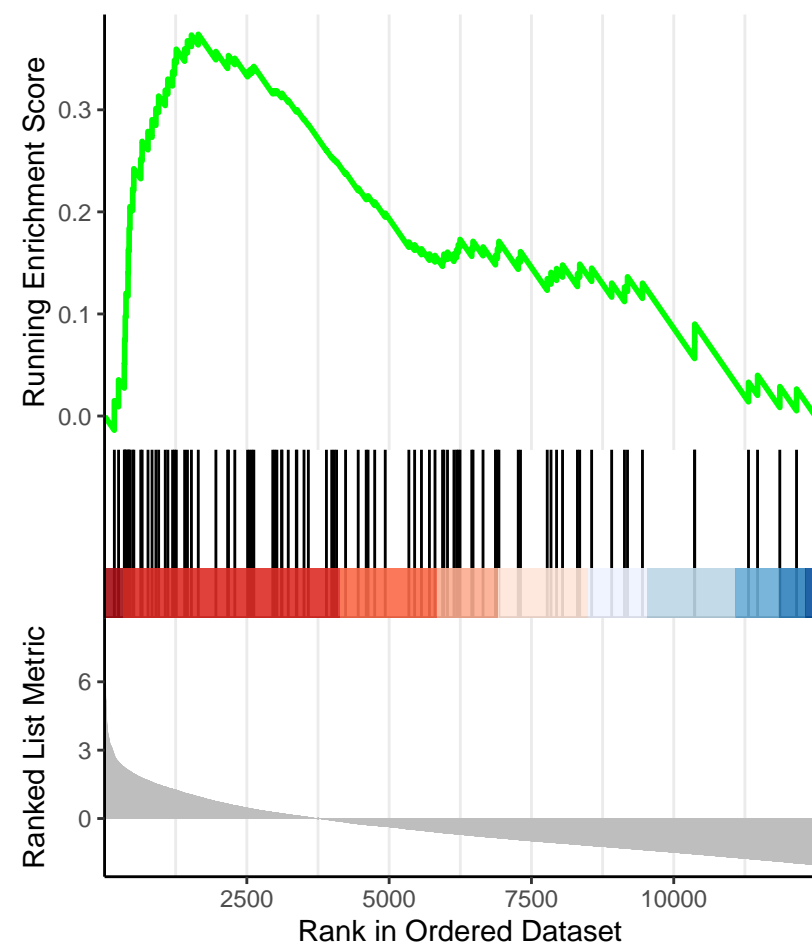

Supplement: Supplementary file 3 [file Presentation2.PDF]
